# Development of a Transcriptional Amplification System Based on the PEG3 Promoter to Target Androgen Receptor-Positive and -Negative Prostate Cancer Cells

**DOI:** 10.3390/ijms20010216

**Published:** 2019-01-08

**Authors:** Pallavi Jain, Pier-Luc Clermont, Francis Desmeules, Amina Zoubeidi, Bertrand Neveu, Frédéric Pouliot

**Affiliations:** 1 CHU de Québec–Université Laval Research Center, Quebec, QC G1R 2J6, Canada; pallavi.jain87@gmail.com (P.J.); pier-luc.clermont.1@ulaval.ca (P.-L.C.); francis.desmeules@hotmail.ca (F.D.); bertrand.neveu@crchudequebec.ulaval.ca (B.N.); 2 Department of Urologic Sciences, Faculty of Medicine, University of British Columbia, Vancouver, BC V5Z 1M9, Canada; azoubeidi@prostatecentre.com; 3 Vancouver Prostate Centre, Vancouver, BC V5Z 1M9, Canada; 4 Department of Surgery (Urology), Faculty of Medicine, Laval University, Quebec, QC G1R 2J6, Canada

**Keywords:** prostate cancer, neuroendocrine prostate cancer (NEPC), progression elevated gene-3 (PEG3) promoter, castration-resistant prostate cancer (CRPC), bioluminescence

## Abstract

Localized prostate cancer (PCa) is often curable, whereas metastatic disease treated by castration inevitably progresses toward castration-resistant PCa (CRPC). Most CRPC treatments target androgen receptor (AR) signaling. However, not all CRPC cells rely on AR activity for survival and proliferation. With advances in immunotherapy and fluid biopsies for cancer management, expression systems specific for both AR-positive and -negative PCa are required for virus-based vaccines and cell imaging. To target both AR-responsive and non-responsive cells, we developed a three-step transcriptional amplification (3STA) system based on the progression elevated gene-3 (*PEG3*) promoter named *PEG3AP1*-3STA. Notably, we report on different genetic modifications that significantly improved PEG3 promoter’s strength in PCa cells. Adenoviruses incorporating *PEG3* promoter with and without transcriptional amplification systems were generated. The potential of *PEG3AP1*-3STA to target PCa cells was then evaluated in vitro and in vivo in androgen-responsive and non-responsive PCa cell lines. *PEG3AP1*-3STA was shown to be active in all PCa cell lines and not regulated by androgens, and its activity was amplified 97-fold compared to that of a non-amplified promoter. The *PEG3AP1*-3STA system can thus be used to target advanced AR+ and AR− cells for imaging or immunovirotherapy in advanced PCa.

## 1. Introduction

Prostate cancer (PCa) represents the most frequent non-cutaneous malignancy and accounts for a third of all cancer-related deaths in men [1]. Treatment of localized PCa by surgery or radiation therapy is initiated with curative intent and is often successful [2]. However, a non-negligible proportion of men develop recurrence following primary treatment or will present with metastatic disease, rendering them unsuitable candidates for localized therapy [3]. These patients are thus treated with androgen-deprivation therapy (ADT), which elicits a significant response in the majority of cases [4]. Unfortunately, these patients eventually relapse and develop lethal castration-resistant prostate cancer (CRPC) [5].

While it is clear that some CRPC cells can sustain androgen receptor (AR) activity despite low testosterone levels, a number of studies have demonstrated that castration resistance can occur through AR-independent mechanisms [5]. For example, CRPC cells can activate anti-apoptotic pathways that can enable cell survival despite the absence of AR expression [6]. Another emerging AR-independent adaptive resistance mechanism is neuroendocrine transdifferentiation [7] through which prostate adenocarcinoma cells undergo a complete phenotypic change to acquire characteristics of small cell carcinoma, thereby giving rise to neuroendocrine prostate cancer (NEPC) [8]. Because NEPC is AR-negative, it is intrinsically resistant to castration and does not increase the serum levels of prostate-specific antigen (PSA), an AR-regulated clinical biomarker [8]. Accordingly, the pathogenesis of castration resistance can be caused by an increase in the number of AR-independent cells, including NEPC cells [9].

To improve prognoses and the response to treatment in castrated patients, a number of promising strategies based on molecular targeting have been developed [10]. Recent advances have provided personalized immunotherapy by incorporating these targets in viral vectors such as PROVENGE [11]. Viral vectors have gained interest in providing tissue-specific therapy, as they are easily engineered and can carry large amounts of genetic material, thereby generating significant amount of clinical applications. Genetically engineered viral vectors expressing PCa-specific markers can be used not only for immunotherapy but also for imaging when combined with the positron emission tomography reporter gene sr39tk [12]. Indeed, when radiotracer 18F-FHBG is administrated, sr39tk-specific expression can be imaged in vivo by positron emission tomography, a clinically available technology [13,14].

Our laboratory has developed the three-step transcriptional amplification (3STA) system, a powerful tool that enables the amplification of promoter transcriptional activity by up to a hundredfold [15]. One promising promoter to couple with the 3STA system is the rat promoter named progression elevated gene 3 (*PEG3*), which has been extensively studied for its important activity in different types of cancers, including PCa [16,17,18]. We thus report on the development and optimization of our *PEG3AP*1-3STA system, which may be deployed in different clinical settings for immunotherapy, cell imaging, or gene therapy. Notably, we outline genetic engineering modifications that significantly improve *PEG3*-driven transcriptional output and we show that *PEG3AP1*-3STA is highly active in PCa cells, including AR-independent and NEPC-like cell lines.

## 2. Results

### 2.1. PEG3AP1-3STA Is a Highly Sensitive Amplification System

**Figure 1:**
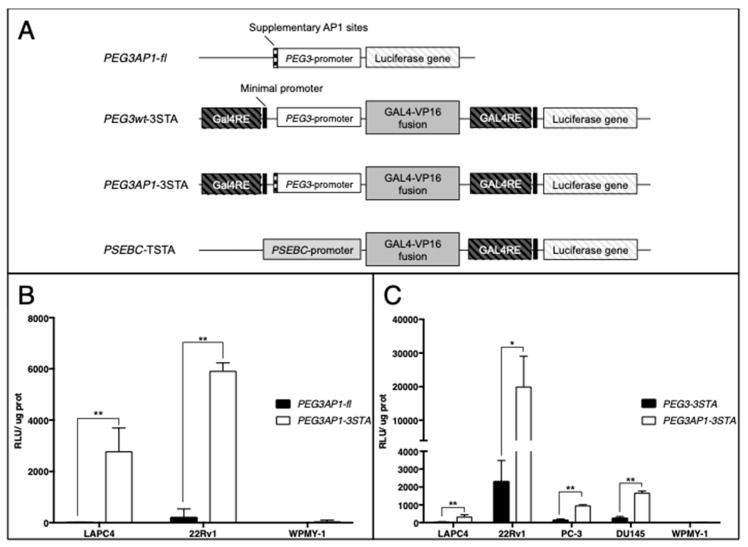
Transcriptional activity of modified progression elevated gene-3 (PEG3) promoter constructs. (**A**) Scheme of non-replicative reporter adenoviral constructs. (**B**) Amplification of *PEG3AP1* promoter by means of the 3STA system. LAPC4 (prostate cancer (PCa)), 22Rv1 (PCa), and WPMY-1 (normal human fibroblastic prostate) cells were transduced with either *PEG3AP1*-fl or *PEG3AP1*-3STA adenovirus at 5 MOI for 72 h. Firefly luciferase (fl) activity was then assayed. The relative luciferase activity was normalized by protein content in each well. Data represent triplicate ± SD. (**C**) Impact of supplementary AP1 sites in PEG3 promoter. LAPC4, 22Rv1, PC-3, DU145 (all PCa), and WPMY-1 (normal human fibroblastic prostate) cells were transduced with either *PEG3*-3STA or *PEG3AP1*-3STA adenovirus at 5 MOI for 72 h. Firefly luciferase activity was then assayed. The relative luciferase activity was normalized by protein content in each well. Data represent triplicate ± SD, * *p* < 0.05, ** *p* < 0.01.

In this study, we cloned the *PEG3* promoter region in the two- or three-step transcriptional amplification (TSTA or 3STA) system to obtain the *PEG3*-3STA. The two-step transcriptional amplification system (TSTA) has three components: a specific promoter, an activator (Gal4VP16), and a reporter. GAL4-VP16 is a fusion protein containing a binding domain (GAL4) and an activation domain (VP16). The binding domain GAL4 specifically binds to GAL4 response elements, then the VP16 domain leads to higher transcriptional activation of the promoter. Specificity of the TSTA system is ensured by the promoter, while its sensitivity is ensured by the amplification system, which can amplify the promoter activity up to 800-fold [12,19]. In the 3STA, an additional Gal4 response element is inserted upstream of the promoter driving the TSTA, which enhances the amplification provided by the TSTA system up to 10-fold [15]. Figure 1A presents a schematic representation of the various adenoviruses constructed for this study. *PEG3* promoter has been shown to have an essential binding site required for its activity, known as AP1, which is also associated with PCa aggressiveness [20]. To further improve the reporter gene signal in PCa cells, two AP1 sites upstream of the *PEG3* promoter sequence were added in the *PEG3*-3STA to obtain the *PEG3AP1*-3STA system (Figure 1A).

First, we investigated whether the 3STA system could increase the transcriptional activity of the *PEG3AP1* promoter (*PEG3AP1*-3STA), compared to *PEG3AP1* alone (PEG3AP1-fl). As expected, the signal was highly amplified in *PEG3AP1*-3STA in two PCa cell lines (LAPC4 and 22Rv1) but not in normal human fibroblastic prostate cells (WPMY-1), indicating that the 3STA system increased transcriptional output specifically in PCa cells (Figure 1B). We then proceeded to validate that the addition of AP1 sites could increase the reporter signal, compared to that of a wild-type promoter, in different PCa cell lines. In the four PCa cell lines investigated, the addition of AP1 sites was found to increase promoter activity (Figure 1C). Here also, no detectable activity was observed in the WPMY-1 cells with either the wild-type promoter or the AP1-modified *PEG3* promoter (Figure 1C). Furthermore, the activity of P*EG3AP1*-3STA compared to that of *PEG3*-3STA increased by 4.9-fold in less aggressive AR+ cells (LAPC4) to 24-fold in more aggressive PCa cells (DU145) (Figure 1C). As these combined data showed that the *PEG3AP1*-3STA system displayed the highest reporter activity, it was thus used for all of the subsequent experiments.

### 2.2. PEG3AP1-3STA Is an Androgen-Independent System

**Figure 2:**
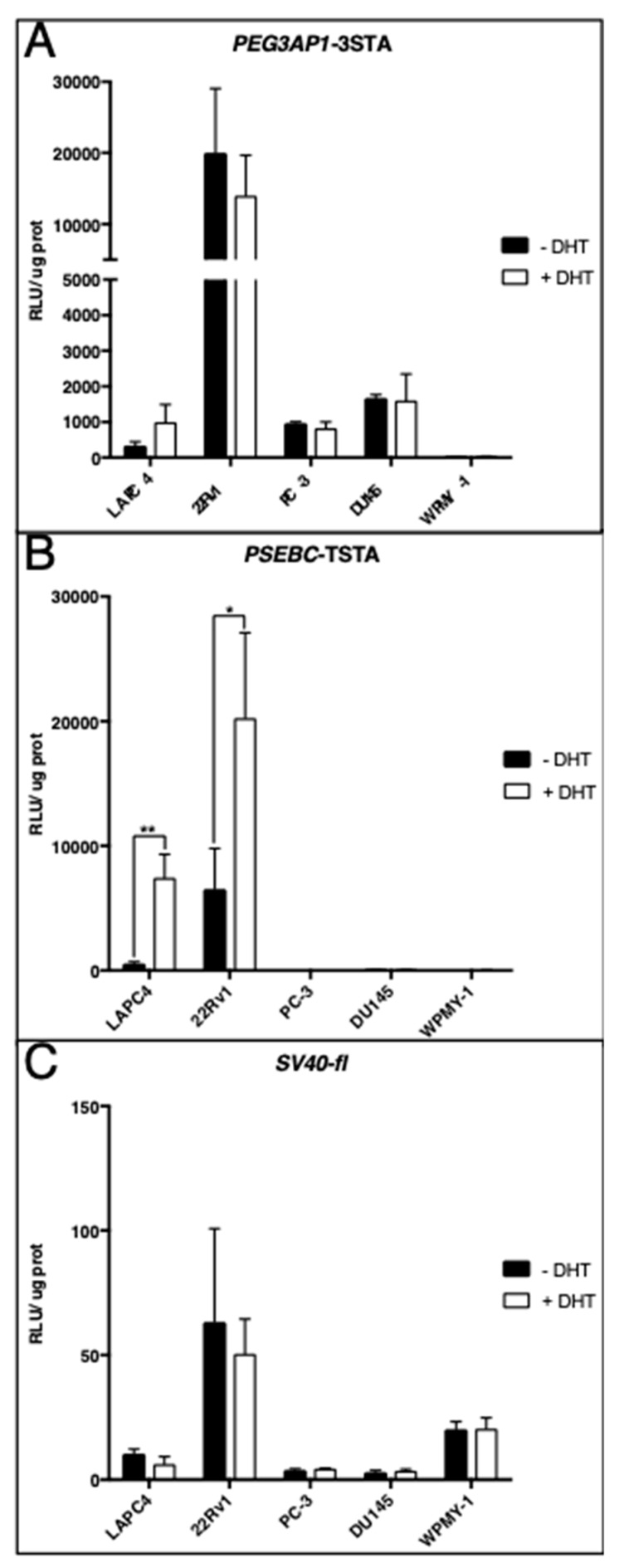
The *PEG3AP1*-3STA system was not androgen-dependent. LAPC4, 22Rv1, PC-3, DU145 (PCa), and WPMY-1 (normal human fibroblastic prostate) cells were transduced with (**A**) *PEG3AP1*-3STA; (**B**) *PSEBC*-TSTA; or (**C**) SV40-fl adenovirus at 5 MOI in the presence or absence of DHT for 72 h. Firefly luciferase activity was assayed. The relative luciferase activity was normalized by protein content in each well. Data represent triplicate ± SD, (* *p* < 0.05, ** *p* < 0.01).

Late-stage PCa becomes resistant to ADT due to several mechanisms either dependent or independent of AR activity. Thus, to target CRPC, it was necessary to develop a system that was not regulated by androgens. To determine whether *PEG3AP1*-3STA activity was modulated through AR signaling, we cultured PCa cells in the presence or absence of dihydrotestosterone (DHT), a potent AR agonist. Figure 2A shows that *PEG3AP1*-3STA was active at a basal level in various AR+ (LAPC4 and 22Rv1) and AR− cell lines (PC-3 and DU145). Of importance is that *PEG3AP1*-3STA activity was not significantly altered in DHT-supplemented media, compared to that observed in androgen-free media, indicating that *PEG3AP1*-3STA was not androgen-regulated (Figure 2A).

To ensure that these conditions were representative of the anticipated AR activity, we performed additional controls with the androgen-responsive PSA-derived *PSEBC* promoter coupled with the TSTA system (*PSEBC*-TSTA) [21]. As expected, *PSEBC*-TSTA was active solely in AR+ cell lines (LAPC4 and 22Rv1), with no detectable activity in AR− cell lines (PC-3 and DU145, Figure 2B). Furthermore, *PSEBC*-TSTA activity was significantly increased in the AR+ cells supplemented with DHT (Figure 2B). Finally, when the activity of a ubiquitous promoter such as *SV40* was analyzed, a detectable signal was observed in all cell lines, including normal prostatic stromal cells (WPMY-1). Because *SV40*-fl (but not *PEG3AP1*-3STA) was active in normal cells, the results show that *PEG3AP1*-3STA was specific to malignant prostatic cells (Figure 2C).

### 2.3. PEG3AP1-3STA Is Active in Both Adenocarcinoma and Neuroendocrine-Like Cell Lines

**Figure 3:**
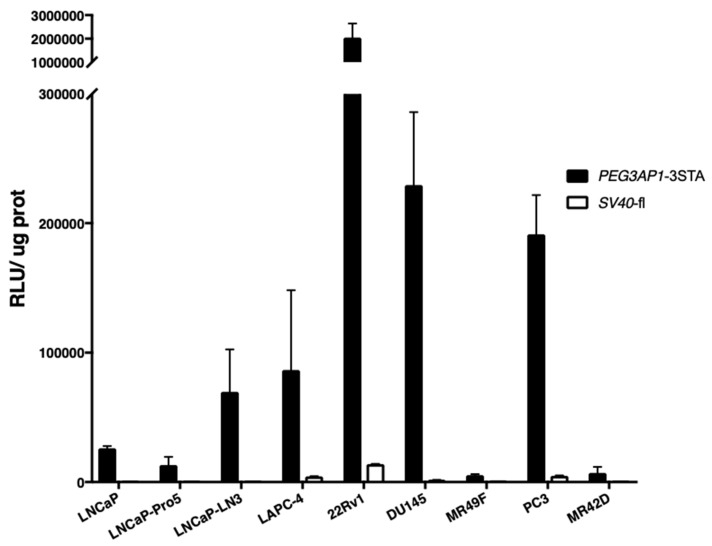
Activity of *PEG3AP1*-3STA in a wide spectrum of prostate malignancies. *PEG3AP1*-3STA relative luciferase activity was quantified in epithelial cells that were castration-sensitive (LNCaP, LNCap-Pro5, LNCaP-LN3, and LAPC4) and castration-resistant (22Rv1, DU145, and MR-49F), as well as in cells exhibiting a neuroendocrine-like phenotype (PC-3 and MR-42D). Compared to the constitutively active promoter *SV40*, *PEG3AP1*-3STA displayed significantly elevated activity in all of the PCa cell lines tested. The relative luciferase activity was normalized by protein content in each well. Data represent triplicate ± SD.

Because NEPC cells are androgen-independent, we hypothesized that *PEG3AP1*-3STA could potentially be active in NEPC. We therefore used epithelial cell lines that were either castration-sensitive (LNCaP, LNCap-Pro5, LNCaP-LN3, and LAPC4), castration-resistant (22Rv1, DU145, and MR-49F), or exhibiting a neuroendocrine-like phenotype (PC-3 and MR-42D) [22,23,24]. Luciferase activity was quantified in these cells following adenoviral transduction with *PEG3AP1*-3STA and *SV40*-fl, a constitutively active promoter used as a positive control (Figure 3). We observed that *PEG3AP1*-3STA generated an 11- to 293-fold higher signal when compared to that of *SV40* in all of the cell lines analyzed. Furthermore, *PEG3AP1*-3STA recorded the highest activity in CRPC cells (22Rv1, DU145) and neuroendocrine-like cells (PC-3). These results indicate that contrary to PSEBC, which is active only in AR positive cells, the *PEG3AP1*-3STA was active in a wide variety of PCa cells with heterogeneous origins and drug sensitivities.

### 2.4. PEG3AP1-3STA Is Active in Heterogeneous PCa Cell Populations within AR+ Cell Lines

**Figure 4:**
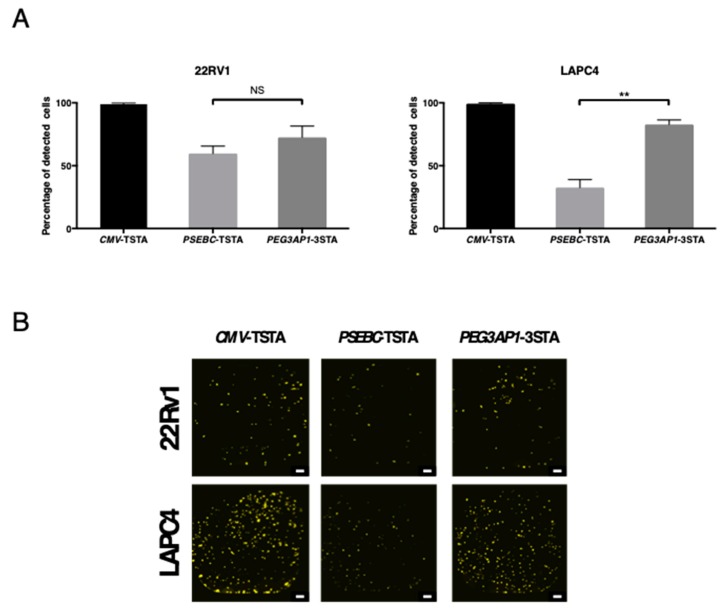
The *PEG3AP1*-3STA system targeted heterogeneous PCa cell populations in AR+ cell lines more efficiently than did *PSEBC*-TSTA. 22Rv1 and LAPC4 GFP-expressing cells were transduced with *CMV*-TSTA, *PSEBC*-TSTA, or *PEG3AP1*-3STA adenovirus. (**A**) Percentage of detected cells (number of luciferase-positive cells ÷ number of GFP-expressing cells) × 100. Significant differences are indicated by asterisks (** *p* < 0.01). “NS” indicates no significant difference between samples. (**B**) Representative images of bioluminescence microscopy. Seventy-two hours after transfection, bioluminescence microscopy imaging was performed. Scale bar represents 200 μm.

We have previously shown that a prostate cancer cell line contains a heterogeneous cell population [21]. To evaluate the potential of the PEG3-3STA system to detect PCa cells despite the established intra-cell line heterogeneity, we calculated the percentage of cells detected with CMV-TSTA, PSEBC-TSTA, or PEG3-3STA adenovirus in 22Rv1 and LAPC4 cells. The three adenoviruses detected 98.9, 59.7, and 72.6% of 22Rv1-GFP cells and 99.3, 32.6, and 82.8% of LAPC4-GFP cells, respectively (Figure 4).

### 2.5. PEG3AP1-3STA Provides a Quantifiable Signal In Vivo That Is Higher Than That of Positron Emission Tomography (PET) Imageable PSEBC-TSTA

**Figure 5:**
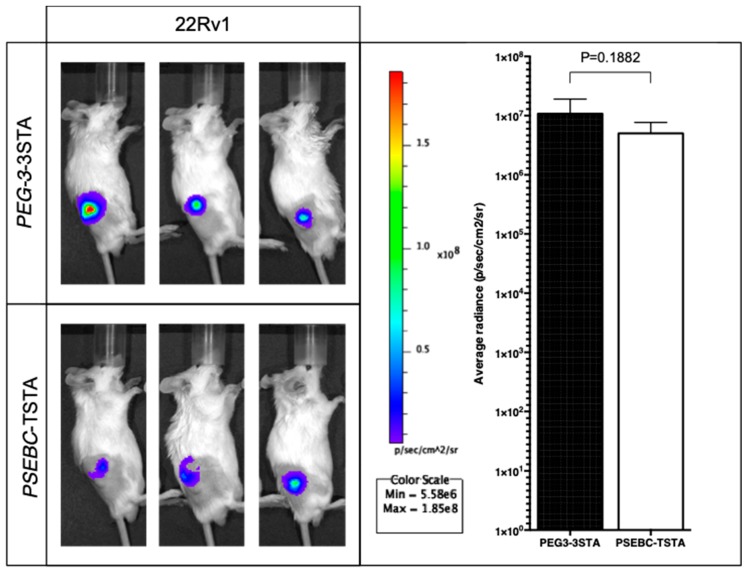
Bioluminescence emitted by the *PEG3AP1-3STA* system was detected and quantified in vivo. 22Rv1 cell subcutaneous xenografts were generated in SCID beige mice. Following tumor growth and mice randomization for tumor volume, 10^8^ infectious viral particles (ivp) of either *PEG3AP1*-3STA or *PSEBC*-TSTA were injected intratumorally. After 96 h, reporter activity was assessed by bioluminescence. Average photon emission per tumor was measured in 22Rv1 mouse xenografts (*n* = 3). No statistically significant difference was observed between the *PEG3AP1*-3STA and *PSEBC*-TSTA firefly luciferase activity in the two cell lines tested (*p* = 0.1882).

We tested the translational potential of the *PEG3AP1*-3STA system in vivo in mice models. 22Rv1 xenografts were transduced with different adenoviruses and subsequently imaged by means of the IVIS Spectrum imaging system. First, the signal elicited by *PEG3AP1*-3STA was strictly restricted to PCa xenografts, with no evidence of non-specific activity (Figure 5). Moreover, luciferase activity generated by *PEG3AP1*-3STA was slightly higher than that by the well-established and functional *PSEBC*-TSTA system (Figure 5, *p* = 0.1882) [21]. As *PSEBC*-TSTA was shown to provide a reporter signal high enough to be detected by a clinical PET apparatus in immune-competent canines [12] or lymph node micrometastases by micro-PET in mice, these results indicate that *PEG3AP1*-3STA activity was sufficient for PET imaging.

## 3. Discussion

In this article, we report the development of a novel transcriptional system, named *PEG3AP1*-3STA, to drive the expression of reporter gene firefly luciferase in PCa cells following adenoviral transduction. While the wild-type *PEG3* promoter is known to be active in many cancers, including PCa cells [17,25], to allow for PET-imaging, the 3STA system can significantly increase the sensitivity of the reporter gene while maintaining promoter specificity, as previously reported with prostate cancer-specific promoter PCA3 [15]. Thus, *PEG3AP1*-3STA represents a new and highly translational tool to target prostate cancer heterogeneous cell populations for imaging or immunotherapy [21].

Currently, clinicians continue to rely heavily on the serum levels of PSA, an androgen-regulated protein, to monitor disease progression [26]. PSA promoter is mainly regulated through AR in the early stages of PCa. However, it has been demonstrated that progression following several lines of systemic therapy may occur with no detectable rise in PSA, notably due to the proliferation of AR-independent subpopulations [27]. It is therefore of prime importance to develop systems that specifically target PCa that is AR-dependent, AR-independent, and ideally NEPC. Our experiments in the presence or absence of DHT also show that the transcriptional competency of *PEG3AP1*-3STA was independent of androgenic regulation. Consistent with these results, we demonstrate that *PEG3AP1*-3STA activity was elevated in both AR-positive and AR-negative cells, including NEPC-like cell lines. Moreover, in contrast to ARE-driven systems, *PEG3AP1*-3STA is fully active in the heterogeneous spectrum of prostate malignancies.

The clinical translation possibilities of this system are numerous. The most promising application constitutes its use as a driver of cytotoxic viruses for tumor-specific antigen release as part of an immunotherapeutic approach. For example, oncolytic viruses have been used for a number of emerging immunotherapeutic strategies based on the expression of immune-modulating cytokines. These cytokines may also be placed under the control of *PEG3AP1* [28] to provide a new and useful transcriptional system that can be employed in the clinic. Furthermore, this system could potentially be used in novel therapies since systemic treatment with the prodrug ganciclovir produces a toxic metabolite only in cells expressing HSV1-sr39tk [29,30]. Indeed, the gene HSV1-sr39tk could be placed under the control of the PEG3-3STA system. Thus, only cells that express the suicide gene HSV1-sr39tk could convert the ganciclovir into its cytotoxic form and trigger apoptosis. As a result, this type of therapy could specifically target PCa cells in which PEG3AP1-3STA is active.

Another major clinical translational potential of our innovative system lies in the detection of CTCs. Recent findings have shown that CTC number plays a critical role in determining the stage and aggressiveness of PCa [31]. CTC number has also been shown to be a promising prognostic marker of drug response in patients both pre- and post-therapy [31,32,33]. However, these existing CTC isolation technologies remain dependent on EpCAM or some epithelial biomarker expression. These technologies do not account for the CTCs that lose, down-regulate, or lack EpCAM expression, and would fail to enrich an important subpopulation of CTCs. In a recent study, replication-competent oncolytic adenoviruses were used to detect PCa CTCs driven by PSA/PSMA regulatory elements. Existing systems have been limited by their sensitivity and by the toxicity associated with the use of replicative adenoviruses [34]. Previously, we showed PCA3 to be a high PCa-specific promoter with very weak promoter activity. A PCA3-driven 3STA system was found to enhance reporter gene expression yet continued to show weak activity in more aggressive PCa cell lines [15]. In another study, we demonstrated that a PSEBC-driven amplification system was capable of detecting cancer cells from body fluids such as blood [21]. Similarly, the *PEG3AP1*-3STA system could complement current PCA3 or PSA assays by detecting circulating tumor cells (CTCs) and providing a more comprehensive assessment of disease monitoring. *PEG3AP1*-3STA may not only provide higher sensitivity but also reduce the toxicity due to the use of non-replicative adenoviruses.

Despite the proposed applications and characterization of the *PEG3AP1*-3STA system, this study does have limitations. First, we acknowledge that we did not directly image tumors using a clinical PET apparatus but only compared the *PEG3AP1*-3STA signal and that of *PSEBC*-TSTA, which has already been shown to generate a signal high enough to be detected by clinical PET. Second, while we recognize that non-replicative adenoviruses are not the most efficient gene delivery methods for immunotherapeutic vaccines, the advantage of the *PEG3AP1*-3STA system is that it can be rapidly cloned in replicative virus genomes to improve its efficacy for therapeutic purposes [35].

## 4. Materials and Methods

### 4.1. Cloning and Virus Production

Adenoviral plasmids were constructed as previously described [15,21]. Briefly, *PEG3* promoter (1477 to 1940 bp of Genbank accession number AF351130) was PCR-amplified from rat genomic DNA. Two AP1 sites (TGACTCA) were added by PCR at the 5′ position of the *PEG3* promoter to obtain pENTR-L1L2-*PEG3AP1*-fl. Plasmid pENTR-L1L2-*PEG3AP1*-fl was subcloned into pAd-pL-DEST by LR cloning (LR Clonase II Plus, Life Technologies, Burlington, ON, Canada) to obtain the *PEG3AP1*-fl adenovirus. The same sets of primers with or without supplementary AP1 sites were used for the construction of *PEG3*-3STA and *PEG3AP1*-3STA adenoviruses using the MultiSite Gateway^®^ Pro 2.0 kit (Life Technologies), as previously described [15]. *PSEBC* promoter, a chimeric *PSA* promoter, was cloned into a TSTA system, as previously described [15,36,37]. The adenoviral plasmids were then transfected into 293A cells for non-replicative adenovirus production. The adenoviruses were purified by means of the Adeno-X Maxi Purification kit (Clontech, Mountain View, CA, USA) and their titers were determined using the Adeno-X™ Rapid Titer Kit (Clontech).

### 4.2. Cell Culture

22Rv1, LNCaP, LNCaP-Pro5, LNCaP-LN3, MR-49F, and MR-42D (prostate cancer cell lines) were cultured in RPMI-1640 media containing 10% fetal bovine serum (FBS). LAPC4 (prostate cancer cell line) and WPMY-1 cells (normal human fibroblastic prostate) were cultured in DMEM media containing 10% FBS. DU145 (prostate cancer cell line) was cultured in EMEM media containing 10% FBS. LAPC4 and DU145 cells were kindly provided by Dr. C. Sawyers and Dr. L. Old, respectively. MR-49F and MR-42D cells were produced by Dr. A. Zoubeidi’s laboratory [38], while LNCaP, LNCaP-Pro5, and LNCaP-LN3 were provided by Dr. C. Guillemette. Other cell lines were obtained from ATCC. Mycoplasma testing was performed using the MycoAlert Mycoplasma Detection kit (Lonza, Basel, Switzerland).

### 4.3. Bioluminescence Quantification In Vitro

22Rv1, LNCaP, LNCaP-Pro5, LNCaP-LN3 MR-49F, MR-42D, LAPC4, PC-3, DU145, and WPMY-1 cells were seeded in 24-well plates. Twenty-four hours later, cells were transduced with indicated adenoviruses at a multiplicity of infection of 5. To assess androgen responsiveness, all of the cells were cultured in their respective medium supplemented with 10% charcoal-stripped FBS with or without the addition of 10 nM DHT. Seventy-two hours after treatment, luciferase assays were performed to measure luciferase activity. Briefly, cells were lysed using a passive lysis buffer (Promega, Madison, WI, USA) and luciferase activity was measured by means of either Luminoskan Ascent (ThermoFisher Scientific, Ottawa, ON, Canada) or TriStar^2^ (Berthold Technologies, Oak Ridge, TN, USA) following the addition of D-luciferin, as stated in the Promega luciferase assay system. Relative luciferase activity (RLU) was normalized to total protein amount in each well (RLU = RLU ÷ μg of protein). Protein content was determined by adding 250 μL of Bradford reagent (ThermoFisher Scientific) to 3 μL of total lysate. Absorbance of the corresponding lysate was then read at 595 nm using an Infinite F50 absorbance microplate reader (TECAN, Mannedorf, Switzerland).

### 4.4. Bioluminescence Microscopy Detection

Bioluminescence microscopy was performed using an Olympus LV200 (Olympus, Tokyo, Japan) microscope designed for luminescence imaging, transmitted brightfield, and transmitted fluorescence imaging. GFP-expressing 22RV1 and LAPC4 cells were seeded in a 384-well black plate (250 cells/well). Twenty-four hours later, the cells were transduced with either *CMV*-TSTA, *PSEBC*-TSTA, or *PEG3*-3STA adenovirus (10^5^ ivp/well). Bioluminescence imaging was performed 72 h post-transduction with 3.5 mM of D-luciferin (Caliper Lifesciences, Hopkinton, MA, USA) using a 40× (20 s of exposure per FOV) objective. The percentage of detected cells was defined as the number of bioluminescent over biofluorescent cells (GFP-positive cells) multiplied by 100.

### 4.5. Bioluminescence Detection in Prostate Cancer Xenografts

Two million 22Rv1 cells mixed in Matrigel (354262, Fisher Scientific, Ottawa, ON, Canada) were injected subcutaneously into the flanks of Fox Chase SCID beige mice (code 250, Charles River Canada, St-Constant, QC, Canada). Tumors were grown for 32 days following intra-tumoral injection of the adenoviruses (10^8^ ivp) in phosphate buffered saline (PBS). Bioluminescence was quantified 96 h later following intraperitoneal injection of D-luciferin with a Xenogen IVIS apparatus (Caliper Lifesciences, Woodbridge, ON, Canada) and is reported as p/s/cm^2^. Animal experiments were approved by the Laval University Animal Care Committee (2010128-3, 1st July 2011).

### 4.6. Statistical Analysis

The tatistical analyses were performed using a two-sided Student *t*-test.

## 5. Conclusions

We developed *PEG3AP1*-3STA, a cancer-specific system that can image heterogeneous populations of PCa cells, including adenocarcinoma and neuroendocrine differentiated cells, with or without androgen receptor expression. This expression system can thus be used to target PCa cells for CTC detection, immunotherapy, or imaging.

## Abbreviations

3STA	Three-step amplification system
ADT	Androgen-deprivation therapy
AR	Androgen receptor
CRPC	Castration-resistant prostate cancer
DHT	Dihydrotestosterone
FBS	Fetal bovine serum
fl	Firefly luciferase
ivp	Infectious viral particules
MOI	Multiplicity of infection
NEPC	Neuroendocrine prostate cancer
PBS	Phosphate buffered saline
PCa	Prostate cancer
PEG3	Progression elevated gene-3
PET	Positron emission tomography
PSA	Prostate-specific antigen
RLU	Relative luciferase activity
TSTA	Two-step transcriptional amplification system
